# Update of: Marx Delaney et al., Improving Adherence to Essential Birth Practices Using the WHO Safe Childbirth Checklist With Peer Coaching: Experience From 60 Public Health Facilities in Uttar Pradesh, India

**DOI:** 10.9745/GHSP-D-18-00064

**Published:** 2018-03-21

**Authors:** Megan Marx Delaney, Pinki Maji, Tapan Kalita, Nabihah Kara, Darpan Rana, Krishan Kumar, Jenny Masoinneuve, Simon Cousens, Atul A Gawande, Vishwajeet Kumar, Bhala Kodkany, Narender Sharma, Rajiv Saurastri, Vinay Pratap Singh, Lisa R Hirschhorn, Katherine EA Semrau, Rebecca Firestone

**Affiliations:** a Ariadne Labs, a Joint Center between Brigham and Women's Hospital and the Harvard T.H. Chan School of Public Health, Boston, MA, USA.; b Population Services International, Lucknow, Uttar Pradesh, India.; c London School of Hygiene & Tropical Medicine, London, UK.; d Department of Health Policy and Management, Harvard T.H. Chan School of Public Health, Boston, MA, USA.; e Department of Surgery, Brigham and Women's Hospital, Boston, MA, USA.; f Community Empowerment Lab, Lucknow, Uttar Pradesh, India.; g Jawaharlal Nehru Medical College, Karnataka, India.; h Feinberg School of Medicine, Northwestern University, Chicago, IL, USA.; i Department of Medicine, Harvard Medical School, Boston, MA, USA.; j Division of Global Health Equity, Department of Medicine, Brigham and Women's Hospital, Boston, MA, USA.; k Population Services International, Washington, DC, USA.

See updated article.

In the article “Improving Adherence to Essential Birth Practices Using the WHO Safe Childbirth Checklist With Peer Coaching: Experience From 60 Public Health Facilities in Uttar Pradesh, India” by Megan Marx Delaney, Pinki Maji, Tapan Kalita, et al., which appeared in the June 2017 issue (Volume 5, Number 2) of GHSP, the authors assessed birth attendants' adherence to essential birth practices in 60 public health facilities in Uttar Pradesh, India. At the time of publication of this article, we noted at the end of the Abstract field that we would update this article by including a reference to the impact findings of the intervention, which were pending publication in another journal. Those impact findings have now been published in the *New England Journal of Medicine*, so we have updated the related GHSP article accordingly. The overall impact findings were that the intervention had no significant effect on maternal or perinatal mortality or maternal morbidity, despite having positive effects on essential birth practices.

These impact findings are now referenced at the end of the Abstract.

